# Do Your Kids Get What You Paid for? Evaluation of Commercially Available Probiotic Products Intended for Children in the Republic of the Philippines and the Republic of Korea

**DOI:** 10.3390/foods9091229

**Published:** 2020-09-03

**Authors:** Clarizza May Dioso, Pierangeli Vital, Karina Arellano, Haryung Park, Svetoslav Dimitrov Todorov, Yosep Ji, Wilhelm Holzapfel

**Affiliations:** 1 Institute of Biology, College of Science, University of the Philippines Diliman, Quezon City 1101, Philippines; cedioso@up.edu.ph; 2 Advanced Green Energy and Environment Department, Handong Global University, Pohang, Gyungbuk 37554, Korea; karina.aray@yahoo.com (K.A.); hrpark@microbes.bio (H.P.); slavi310570@abv.bg (S.D.T.); 3 Natural Sciences Research Institute, University of the Philippines Diliman, Quezon City 1101, Philippines; pierangeli.vital@upd.edu.ph; 4 HEM Inc., Business Incubator, Handong Global University, Pohang, Gyungbuk 37554, Korea; ysji@microbes.bio

**Keywords:** probiotics, children, label claims, viable count, molecular identification, safety, lactic acid bacteria

## Abstract

A wide range of probiotic products is available on the market and can be easily purchased over the counter and unlike pharmaceutical drugs, their commercial distribution is not strictly regulated. In this study, ten probiotic preparations commercially available for children’s consumption in the Republic of the Philippines (PH) and the Republic of Korea (SK) have been investigated. The analyses included determination of viable counts and taxonomic identification of the bacterial species present in each formulation. The status of each product was assessed by comparing the results with information and claims provided on the label. In addition to their molecular identification, safety assessment of the isolated strains was conducted by testing for hemolysis, biogenic amine production and antibiotic resistance. One out of the ten products contained lower viable numbers of recovered microorganisms than claimed on the label. *Enterococcus* strains, although not mentioned on the label, were isolated from four products. Some of these isolates produced biogenic amines and were resistant to one or several antibiotics. Metagenomic analyses of two products revealed that one product did not contain most of the microorganisms declared in its specification. The study demonstrated that some commercial probiotic products for children did not match their label claims. Infants and young children belong to the most vulnerable members of society, and food supplements including probiotics destined for this consumer group require careful checking and strict regulation before commercial distribution.

## 1. Introduction

The microbiome of infants and young children undergoes dynamic development and is shaped by a combination of factors including the specificity and change in the diet (e.g., breast-feeding to solid food commodities), life environment and socio-cultural conditions. These and other ecological and environmental factors strongly impact the development of the gut microbiome, resulting from the changing microbial balance towards increasing diversity during early life. Disturbance of gut homeostasis by, e.g., antibiotics and opportunistic infections, may result in the development of gastrointestinal disorders such as diarrhea in young children [1,2]. On a global scale, children below three years of age may experience at least one episode of gastroenteritis every year [3]. One approach in preventing or treating diarrhea by healthcare professionals is through the introduction of beneficial microorganisms or probiotics to stabilize the microbial population in the gastro-intestinal tract (GIT) [4,5]. Probiotics are defined by the Food and Agriculture Organization (FAO) and World Health Organization (WHO) as “live microorganisms which when administered in adequate amounts confer health benefit to the host” [6]. The European Society for Pediatric Gastroenterology, Hepatology and Nutrition (ESPGHAN group) has recommended the use of probiotics in preventing different types of diarrhea in children including acute-infectious diarrhea, antibiotic-associated diarrhea (AAD) and nosocomial or hospital-acquired diarrhea [7,8]. Studies have demonstrated that administration of probiotics could lower the severity and duration of diarrhea among children. The use of probiotics for management of (acute) diarrhea in children is particularly recommended in cases of existing or elevating risks such as selective increase of antibiotic resistance, hospitalization, and dehydration [5]. Some of the common bacterial strains in children probiotics studied for prevention of nosocomial diarrhea include *Lactobacillus rhamnosus* GG (LGG)*, Lactobacillus reuteri* DSM 17938, *Bifidobacterium animalis* BB12 and *Saccharomyces boulardii* [4,9,10]. Yet, Cochrane meta-analyses on the use of probiotics for the treatment of persistent diarrhea in children have indicated only limited success [5,11].

A range of probiotics for children is currently marketed as food commodities, dietary supplements and pharmaceutical preparations [12]. These products are available in different commercial forms such as powders, granules, vials, tablets, capsules and gel capsules. Especially for children, probiotic products have also been commercialized as chewable tablets for easier administration.

According to the WHO definition, probiotics should contain viable microorganisms that, when administered in adequate numbers, confer a health benefit to the host and by implication, these strains should be metabolically active [12]. Health Canada and the Italian Ministry for Health recommended a minimum of 1 × 10^9^ colony forming units (CFU) per serving as an acceptable dose for effective health promotion by a probiotic microorganism [13]. However, for biologically unstable probiotic products lower (strain dependent) viable numbers may be expected to reach the intestinal destination upon consumption [12] due to diverse stress conditions in the gut ecosystem [14]. Two minimum requirements for a microbial strain to be considered as a “probiotic” are recommended by the Special Working Group of the Belgian Superior Health Council. Firstly, the organism should be taxonomically identified at the genus, species and (alpha-numerically) at the strain level. In addition, in vitro physiological characterization by screening tests should give information on its “resistance to gastric acidity, bile acid and digestive enzymes and against potentially pathogenic bacteria” [15].

Although an acceptable scientific definition has been recommended by the WHO, it is still considered as “recommendation” while consensus on a “legal” definition of the term “probiotic” has not been reached yet. Hence, the term is being used commercially, often as advertising strategy, for products that possibly might not meet the minimum requirements for a probiotic [16]. Driven by a constantly growing demand, the assortment and diversity of supplements on the market claimed to be probiotics, is steadily increasing. The global value of the probiotic industry is projected to reach about 78.3 billion US dollar by 2026. The Asia-Pacific region which includes countries such as the Republic of the Philippines (PH) and Republic of Korea (SK), is expected to be “the fastest-growing region in the probiotic market” [17]. Moreover, it appears that probiotic products are already dominating the infant formula market and may reach a projected 76% share of this sector in 2024 [18].

Absent or unclear legal regulations in probiotics market open the way for opportunistic speculations and commercialization of preparations without scientifically proven benefits and/or no final control of the contents. Marketed as food supplements, it appears that most of these probiotic products have no scientifically confirmed therapeutic claims, and, unlike therapeutic drugs, are not strictly regulated. Unfortunately, the lack of global standards may lead to improperly labeled products. Frequently, consumers are confused due to the lack of clear labeling standards for probiotic products [12]. Several studies have shown inconsistencies in the labels of probiotic products, particularly with regards to total viable counts and strain identification [19,20,21].

This study was motivated by the absence both of documented studies evaluating the quality and efficacy of probiotic products intended for children and of strict regulations for probiotics designated as supplements. In these investigations, the authors aimed to specifically evaluate probiotic products intended for children in the PH and SK. Label claims served as reference to the expected viable numbers of probiotic microorganisms present, and their identity as determined by culture dependent and independent methods. In addition, antimicrobial properties and safety were determined for representative strains, while in vitro functionality was assessed on the basis of survival under simulated GIT conditions.

## 2. Materials and Methods

### 2.1. Acquisition of Commercially Available Probiotics

Five different probiotic products intended for children were obtained over the counter from pharmacies in PH, each represented by two different batch or lot numbers. Another five different probiotic products were bought in pharmacies and from online commercial stores in SK. The product specifications, including batch or lot number, expiration dates and label claims of these products were noted. The products were stored according to the prescribed conditions indicated on the label or, if not stated, at room temperature around 25 °C, following pharmacist recommendations. All tested products had a claimed shelf life of at least 6–8 months prior to analysis. The evaluated probiotic products were available in different forms such as tablets, powders, granules, and chewable tablets. For this study, each product was assigned a code using the letters A, B, C, D, E, F, G, H, I and J in order to ensure confidentiality of brand names and manufacturers.

### 2.2. Evaluation of the Cell Viability

#### 2.2.1. Sample Processing

Culture-based techniques were used for determination of viable cell numbers of microorganisms present in each product. Depending on the pharmaceutical form type, appropriate modifications in the analysis were performed to ensure the viability of the cells.

For both powdered and granulated samples, 1 g of each probiotic product was dispensed in 9 mL of sterile 1X phosphate buffer saline solution (PBS, Lonza, Walkersville, MD, USA). For samples in tablet form, each tablet was crushed while still in their original packaging using a sterile mortar and pestle. One gram of the powdered tablets was diluted in 9 mL of the same buffer. For samples in gel capsules, capsules were weighed, dissolved in the corresponding volume of 1X PBS supplemented with 1 g/L of Tween 80 (Duksan Chemicals, Gyeonggi-do, South Korea) and homogenized in a stomacher (Seward Stomacher 400 Circulator, Norfolk, UK) at 200 rpm for 3 min. Powdered, granulated and tablet forms were homogenized by vortexing and all the samples were serially diluted up to either 10^−7^ or 10^−8^ in the same buffer depending on CFU numbers declared on the label.

#### 2.2.2. Enumeration of *Lactobacillus* Species

For enumeration of *Lactobacillus* species, when their presence was indicated on the product specification, 0.1 mL of the product cell suspension was spread on de Man Rogosa Sharpe (MRS) agar (BD Difco, Detroit, MI, USA) and let dry. Only the highest three dilutions were used per sample and plated by duplicate. The plates were incubated for 48 h at 37 °C in anaerobic chamber (Whitley DG250 Anaerobic Workstation, Don Whitley Scientific Limited, Bingley, West Yorkshire, UK). For products indicating *Lactobacillus acidophilus* as part of its formulation, the sample suspensions were plated on Modified Rogosa agar pH 5.5 and incubated under the same conditions following the same approaches. The formulation for one liter of Modified Rogosa agar consisted of 10 g Bacto™ tryptone, 5 g yeast extract (BD Difco), 20 g D-glucose, 6 g KH_2_PO_4_, 1 g Tween 80, 2 g triammonium citrate, 0.575 g MgSO_4_·7H_2_O, FeSO_4_·7H_2_O (Sigma-Aldrich, St. Louis, MO, USA), 0.110 g MnSO_4_·H_2_O (Duksan Chemicals) and 15 g agar (LPS Solution, Daejeon, South Korea). The pH was adjusted to 5.5 using glacial acetic acid (Daejung Chemicals, Siheung, South Korea) prior autoclaving at 121 °C for 10 min to avoid hydrolyzation of agar due to low pH. Based on the colony counts the CFU/g was were determined for each product.

#### 2.2.3. Enumeration of *Bifidobacterium* Species

For products indicating *Bifidobacterium* species on the label, 0.1 mL of each product cell suspension was spread on Bifidobacterium Agar (Cell Bio, Seoul, South Korea). As previously described, the highest three ten-fold dilutions were plated in duplicate. Plates were incubated under anaerobic conditions for 48 h at 37 °C and the CFU/g were determined, as described.

#### 2.2.4. Enumeration of Streptococcus thermophilus

To determine the presence and cell counts of *Streptococcus thermophilus,* 0.1 mL of cell suspensions ranging from 10^−1^ to 10^−4^ dilutions, were spread on M17 Agar (Merck, Darmstadt, Germany) according to Terzaghi [22] and the plates were incubated at 42 °C under aerobic conditions for 24 to 48 h in duplicate. Presence of white colonies with 1–2 mm in diameter were presumed to be representative of *Strep. thermophilus* and were counted to obtain the CFU/g of probiotic product.

#### 2.2.5. Enumeration of *Enterococcus* Species

For probiotics indicating *Enterococcus* species in their product specification, 0.1 mL of the cell suspension of the most appropriate highest three dilutions were spread plated on Enterococcossel Agar (BD Difco) in duplicates. Plates were incubated aerobically for 24–48 h at 37 °C. According to the specifications of the growth medium, brown–black colonies were considered as potential *Enterococcus* species. Results were expressed as CFU/g of probiotic product.

#### 2.2.6. Detection of *Staphylococcus* spp. as Contaminants

The presence of contaminating *Staphylococcus* spp. was confirmed using 3M™ Petrifilm™ Staph Express Count plate (3M, Saint Paul, MI, USA). One mL of the first two dilutions of each product cell suspension was inoculated onto Petrifilms, spread using 3M™ Petrifilm™ spreader (3M) and incubated aerobically at 37 °C. Red-violet colonies were presumed to represent *Staphylococcus* species and were transferred to Tryptic Soy Agar (BD Difco) plates for purification and identification.

### 2.3. Isolation and Purification of Microorganisms from Probiotic Products

Each colony with distinct morphology that grew on the agar plates was streaked onto the same fresh solid media and incubated under anaerobic conditions at 37 °C for 24 to 48 h, except for *Strep. thermophilus* incubated under aerobic conditions at 42 °C. This step was consecutively repeated at least three times until single colonies were observed. Strains were stocked in presence of 30% (*v*/*v*) glycerol, stored at −80 °C and deposited in the HEM Culture Collection (Pohang, South Korea) where strain numbers were given.

### 2.4. Culture-Based Molecular Identification of Microorganisms from Probiotic Products

The obtained pure cultures were identified based on 16S rRNA sequencing analysis after amplification using the universal primers 27F (5′-AGAGTTTGATCCTGGCTCAG-3′) and 1492R (5′ GGTTACCTTGTTACGACTT-3′) from the company Solgent (Daejeon, South Korea). The analysis was performed from single colonies according to the protocol of Solgent. Sequences were aligned and compared with reported sequences available on the NCBI BLAST Database (http://blast.ncbi.nlm.nih.gov/Blast.cgi) for the bacterial identification.

#### Phylogenetic Analysis of Isolated Microorganisms

Phylogenetic trees were constructed using the partial 16S rRNA gene sequences of isolates from each product. The obtained nucleotide sequences were first aligned using MUSCLE [23]. The corresponding alignment was then further trimmed. Then, a neighbor-joining tree was constructed based on the concatenated alignments using MEGA X [24] and 10,000 non-parametric bootstrap replicates to create the optimal tree. The percentage of the bootstrap results was written next to the branch or node [25].

### 2.5. Culture-Independent Metagenomic Analysis of Microorganisms from Probiotic Products

#### 2.5.1. DNA Extraction

Total DNA from the microbial population was extracted from the probiotic product by weighing 0.1 g in a 2 mL tube containing 0.3 g of 0.1 mm zirconium beads and 700 µL of ASL buffer (Qiagen, Hilden, Germany). The mixture was homogenized, and the DNA released from the bacterial cells by 3 min of rigorous minibead beating in Qiagen/Retsch MM300 TissueLyser (Qiagen). Final DNA purification was done following the manufacturer’s protocol using QIAamp DNA Mini Kit (Qiagen) and stool buffer (Qiagen). The purity and concentration of the extracted DNA was checked using the SPECTROstar^Nano^ (BMG Labtech, Ortenberg, Germany) spectrophotometer.

#### 2.5.2. Metagenomic Analysis of Probiotic Products

Only three out of the 10 products in this study were claimed (on the label) to contain only one bacterial species. For the evaluation of products claimed to contain multiple species without culturing, metagenomic analysis is considered a promising method. Due to limitations in resources, two products (A and G), each claimed to contain a mixture of different species, were selected for this assessment; the analysis was performed according to the protocols suggested by Han and Park et al. [26]. For this purpose, 5 ng/µL of the previously isolated genomic DNA was used for the library preparation of each sample according the Illumina 16S Metagenomic Sequencing Library Protocol (Illumina, San Diego, CA, USA). Amplicon primers were obtained from Macrogen (Seoul, South Korea) according to the protocol above and attached to the sample DNA through a PCR reaction specifically targeting the 16S rRNA V3-V4 region. The PCR products were purified using the AMPureXP Beads (Beckman Coulter, Brea, CA, USA) and a 96-Well Ring Magnet Plate T480 (Permagen Labware, Peabody, MA, USA). Dual indices were attached to the samples by using the Nextera XT Index Kit (FC-131-1002, Illumina) and the indexed samples were sequenced on an Illumina Miseq system. The raw data were further analyzed using the MacQIIME [27] 1.8.0 pipeline. Chimeras were eliminated using Usearch 6.1 and sequences were clustered into operational taxonomic units (OTU) at 99% sequence similarity, and taxonomically assigned using Green Genes database.

### 2.6. Safety Assessment of Isolated Strains

#### 2.6.1. Hemolytic Activity of the Isolates

Hemolytic activity of the isolates was determined according to Ji et al. [28]. An 18 h old culture of each strain was transferred onto prepared Blood Agar plates containing 5% (*v*/*v*) defibrillated sheep blood (Synergy Innovation, Seongnam-si, Korea). The plates were incubated at 37 °C for 24 h. Hemolytic activity was noted by checking for either clear zones of hydrolysis (β-hemolysis), a green zone of partial hydrolysis (α-hemolysis) or no clearing (γ-hemolysis) around the colonies. *Bacillus cereus* ATCC 27348 was used as positive control for β-hemolysis, *Escherichia coli* ATCC 25922 for α-hemolysis and *Lactobacillus plantarum* 299v for γ-hemolysis.

#### 2.6.2. Biogenic Amine Production

Amino acid decarboxylase activity was determined according to Bover-Cid and Holzapfel [29] with slight modifications. Eighteen-hour old cultures of evaluated strains were streaked on decarboxylase media containing 1% of each precursor amino acid L-tyrosine (Samchun Chemicals, Seoul, Korea), L-histidine hydrochloride monohydrate (Daejung Chemicals), L-ornithine monohydrochloride (Sigma-Aldrich), and L-lysine monohydrochloride (Samchun Chemicals) respectively. Plates were incubated at 37 °C for 24 h under aerobic or anaerobic conditions depending on the isolates to be tested. Evidence for biogenic amine production was indicated by a change in color of the medium from yellow to purple caused by an increase in pH due to amino acid decarboxylation. *E. coli* ATCC 25922 served as positive control.

#### 2.6.3. Antibiotic Resistance Test

Agar diffusion assay was performed following the guidelines set by the Clinical Laboratory Standards Institute [30]. Isolates were tested against nine antibiotics solutions namely ampicillin sodium salt, gentamicin sulfate salt (Georgiachem, Norcross, GA, USA), erythromycin, tetracycline, streptomycin sulfate salt, chloramphenicol, kanamycin sulfate, clindamycin hydrochloride (Sigma-Aldrich), and vancomycin hydrochloride (Wako Pure Chemical Industries, Osaka, Japan). Prior to testing, isolates were sub-cultured three times in appropriate liquid growth media. Lactic acid bacteria susceptibility medium (LSM) Agar consisted of 90% ISO-sensitive broth (Oxoid, Hampshire, UK) and 10% MRS broth, supplemented with 1.5% agar and adjusted to pH 6.7 using glacial acetic acid. For testing *Bifidobacterium* spp., 0.03% (*w*/*v*) L-cysteine hydrochloride (Samchun Chemicals) was added to LSM [31]. Cells were harvested by centrifugation at 7200× *g*, 4 °C and diluted with 1 M PBS to standardize to approximately 10^7^ CFU/mL of the isolate, and then spot inoculated using a multi-pin inoculator giving a final concentration of 10^5^ CFU/mL per spot. Both types of agar contained two-fold serial dilutions of the antibiotics. Plates containing *Lactobacillus* and *Enterococcus* species were incubated aerobically at 37 °C while *Bifidobacterium* plates were incubated anaerobically at 37 °C. The isolates were also inoculated on plates without antibiotics as a negative control. Growth was checked after 24 h and 48 h. The lowest concentration that completely inhibited growth on an agar plate, disregarding haze that could be caused by the inoculum or single colony, was considered as minimum inhibitory concentration (MIC) value of a strain. The MIC values were compared with the suggested respective breakpoints as recommended by the European Committee on Antimicrobial Susceptibility Testing (EUCAST) [32] and adapted by the European Food Safety Authority [33].

### 2.7. In Vitro Survival of Isolates to Simulated Stomach Duodenum-Passage

The ability of the isolates to survive passage through the human GIT was determined in vitro under conditions physiologically simulating the low pH and bile salts stress of the upper GIT, according to Mathara et al. [34]. A 1% (*v*/*v*) concentration of each isolate was inoculated in 10 mL broth of the corresponding media and incubated under the specified conditions. The cultures were harvested by centrifugation at 10,000× *g* for 5 min and washed with 1X PBS. Then the pellets were resuspended and diluted ten-fold in the same buffer to reach a final concentration of 2 × 10^8^ CFU/mL, determined by measuring the optical density at 600 nm (SPECTROstar^Nano^), and based on previously prepared standard curves.

For the test, one mL of the prepared suspension was added to 9 mL broth at pH 2.5 (adjusted with 5 M HCl) of the corresponding media and homogenized. The mixture was incubated at 37 °C under anaerobic conditions for one hour to simulate the stomach conditions after ingestion of the probiotic product.

In the next step, 4 mL of 10% (*w*/*v*) Oxgall (BD Difco) and 17 mL of synthetic duodenal secretion (adjusted to pH 7.4 using HCl and consisting of 6.4 g of NaHCO_3_ /L, 0.239 g of KCl /L and 1.28 g of NaCl /L) were added to the mixtures to expose the bacterial cells to bile salt stress and simulate the small intestine passage. The tubes were incubated for two more hours under the same conditions. The initial viable counts and those after one- and three-hours incubation were determined by plating on the same agar media used for the test and incubated anaerobically at 37 °C for 24 to 48 h and 42 °C for 24 to 48 h aerobically for *Strep. thermophilus*. The survival rates after stomach and duodenum passage were calculated based on the initial counts.

### 2.8. Investigation of Antimicrobial Properties of Isolates

To determine the activity of the isolated strains against selected GIT pathogens, the agar well diffusion assay was performed. Evaluated strains were grown overnight at 37 °C in the appropriate growth medium. Cell-free supernatants were obtained after centrifugation at 7200× *g* for 10 min at 4 °C. The cells were resuspended in 1M PBS and adjusted to approx. 1.0 × 10^7^ CFU/mL based on the absorbance at 600 nm according to the previously prepared standard curves.

Test organisms used in the determination of antagonistic tests were grown overnight on appropriate growth media under aerobic conditions at 37 °C. A volume of 100 µL of the test organism was spread on Brain Heart Infusion (BHI) agar supplemented with 1% agar (BD Difco). Wells were punched in these BHI agar plates and 20 µL of whole cell suspensions or cell-free supernatant were placed in the wells. Plates were incubated upright aerobically at 37 °C for 24 h and the zones of inhibition were measured using following formula:

Zone of inhibition (mm) = total zone of inhibition (mm) − diameter of well (mm).

### 2.9. Statistical Analyses

Results are expressed as standard deviation of replicates. Graphs and statistical analyses (where applicable) were done using the GraphPad Prism software (v. 8.04, GraphPad Software Inc., San Diego, CA, USA). All data are presented as means with standard deviation.

## 3. Results and Discussion

### 3.1. Viable Count Assay

In this study, 10 probiotic products claimed to be suitable for children and obtained from available commercial retails in PH and SK were analyzed. Initially, the basic criterion for assessment was to check the number of viable microorganisms in each product at the time of purchase. The total CFU/g of each product was determined by the sum of the CFU/g of all the media that were used with reference to the product label.

**Figure 1:**
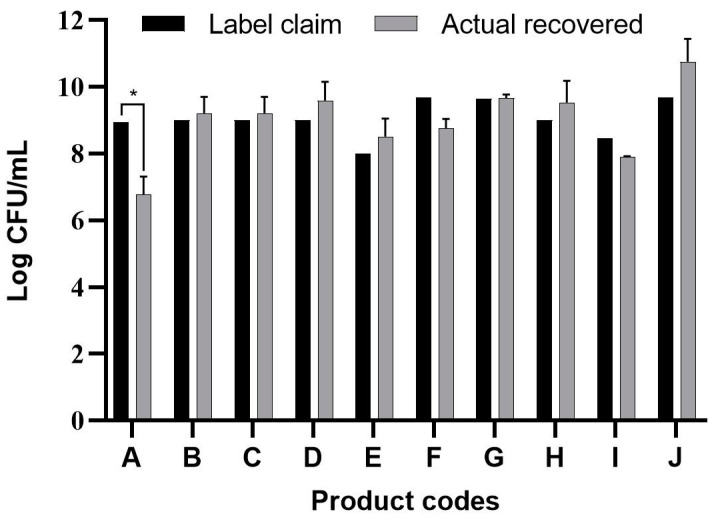
Quantitative label claims versus recovered CFUs from commercial probiotic products intended for children in the Republic of the Philippines (products A–E) and Republic of Korea (products F–J) (* *p* < 0.05).

The results are summarized in Figure 1 and show that significantly lower microbial numbers were recovered from product A as compared to its label claim (*p* < 0.05). Lower viable cell numbers may result in a lower efficacy of the expected (claimed) functions of the product; this may be worsened by a further reduction in viable cell numbers when exposed to the harsh conditions of the human GIT.

### 3.2. Culture-Dependent Molecular Identification

It is the ethical responsibility of the producers to provide consumers with probiotic products that are scientifically proven to be effective and safe. Correct strain identification based on the state-of-the-art technology is one of the requirements of the US Food and Drugs Administration (FDA) and European Food Safety Authority (EFSA) before producing and selling probiotic products. Recent advances in molecular and microbiological techniques have served to improve the reliability of probiotic strain identification and thus prevent confusion [35]. In this study, 16S rRNA sequencing was used for the identification of the isolated microorganisms from the 10 evaluated probiotic products. The use of comparative 16S rRNA sequencing is widely accepted and considered a reliable basis for species identification [36]. While 16S rRNA represents a well-established molecular approach for species level identification, whole genome sequencing (WGS) is regarded as the “gold standard“ for intraspecies (strain level) differentiation [36]. As shown in Table 1, some isolates such as *Bacillus coagulans* from product A have been isolated on different occasions and in different media (e.g., MRS, Rogosa, Enterococossel Agar) and were shown to have the same identity by 16S rRNA sequencing. The constructed phylogenetic tree (Table 1) showed that these *Bac. coagulans* strains (HEM C18, HEM C19, HEM C41, HEM C42, HEM C43, HEM C140) had indeed minor differences in their 16S rRNA nucleotides as shown by the short branch lengths (<0.010). Corresponding to less than 1 nucleotide difference for every 100 nucleotides this suggests that these isolates could be clones of the same strain of *Bac. coagulans*. Hence, only one of these isolates was selected for further characterization (safety and functionality). Similar results were obtained with isolates of *L. rhamnosus* and *L. gallinarum* from product B, as shown in Table 1. Phylogenetic trees of *E. faecium* isolates from products G, I and J, clustered together in the one branch with a bootstrap value of 100, possibly indicating that these isolates were representatives of the same strain. Similar inferences can be made for *L. plantarum* isolates from product H as well as *E. faecium* isolates from product I and *E. faecium* and *L. reuteri* isolates from product J (Table 1). Hence, only one isolate among those with similar species identities were selected for further characterization (safety and functionality).

Table 1 shows the microorganisms listed in the labels of the ten evaluated products in comparison to those actually detected and identified in this study. Microorganisms recovered from products C, D and F fully matched their label claims on species level. For product A, on the other hand, only one (*L. plantarum*) out of 12 claimed species could be confirmed, while the other two recovered (*Bac. coagulans* and *E. faecium*) have not been mentioned on the label. In product B, only four out of the seven indicated species were recovered, considering that the specific strain of *L. casei* could actually be *L. paracasei,* as member of the so-called *L. casei* group [37]. Although phenotypically and genotypically closely related, wrong nomenclature may occasionally be used for *L. casei* strains and it appears that most commercial strains currently distributed are actually representatives of *L. paracasei* [38,39,40]. It is possible that *L. acidophilus* strain from Product B belongs to *L. gallinarum*, a species closely related to *L. acidophilus*, which, on the basis of the 16S rRNA gene, forms a cluster of closely related species with *Lactobacillus crispatus, Lactobacillus helveticus* and *L. gallinarum* [41]. This was also observed in the constructed phylogenetic tree of isolated strains from product B as shown in Table 1 where *L. acidophilus* HEM C16 clustered with the other *L. gallinarum* isolates from the same product.

Although indicated on the labels, the presence of bifidobacteria could not be confirmed in any of the products A, B, E, I and J by the culture-dependent methods applied in this study. On the other hand, *Bifidobacterium breve* was detected in product G instead of *Bifidobacterium longum* and *Bifidobacterium bifidum*, while the presence of *Bif. breve* but not *Bif. longum* could be confirmed for product H. 

Comparing culture-dependent and culture-independent methods for the qualitative detection of bifidobacteria in probiotic products, Masco et al. [44] reported only 70.7% recovery with conventional plating methods as compared to 96.5 % when culture-independent DGGE analysis was used. Misidentification of bifidobacteria, especially in probiotics products intended for children, is considered a “cause for concern for those involved in clinical trials and consumers of probiotic products” [45]. Especially *Bifidobacterium longum* subsp. *infantis* appears to have benefits for the premature intestinal tract superior to those of other bifidobacteria [45].

*Lactobacillus sporogenes* was indicated on the label of product I, however, according to the 7th edition of the Bergey’s Manual of Determinative Bacteriology its correct nomenclature is *Bacillus coagulans* [46]. The use of either incorrect or non-scientific designation for a bacterial strain appears to be a common error. This problem has been reported for several products intended for food and pharmaceutical applications, including probiotics [47]. Such “errors” may lead to confusion and wrong interpretation of strain-related claims. Accurate identification is essential for the correct handling and promotion of a probiotic strain. This has special importance for companies dealing with the production, commercialization and distribution of live bacterial (probiotic) products.

The fluctuations in the claimed microbial consortia detected in the study may be linked to the culture-dependent methods applied. One of the limitations of culture-dependent approaches is that some cells may be in a viable but not culturable (VBNC) state. Therefore, more than one approach needs to be applied in the critical evaluation of commercial products. The results from the culture-independent metagenomic analysis are presented in Table 2 and discussed in the next section. Competition for nutrients may inhibit the growth of some strains and may be a further explanation for the non-recovery of microorganisms mentioned in the product label. However, in this case, it might pose a challenge to property claims of a given (strain/species) combination for commercial application. This was demonstrated in a study reporting on the decline of one *Bif. longum* strain when co-cultured with other probiotic strains [48].

*Enterococcus* spp. were detected but not declared in four products: C (*E. durans*), E (*E. faecalis*), H and I (both *E. faecium*), while E. faecium (and not *E. faecalis*) was detected in product A. The presence of *E. faecium* was confirmed for product J, but, in addition, *E. durans* was also found. Contamination of probiotic products by enterococci, and particularly *E. faecium*, seems to occur occasionally. Examples were reported for some probiotic products intended for the Italian and European market [35]. Although the safety of *E. faecium* is still debated, this species is reported to harbor safe strains (e.g., *E. faecium* SF68) with no virulence factors, some of which have been commercially available for several decades and with no negative surveillance reports. In fact, the safety of *E. faecium* strain SF68, deposited as strain NCIMB 10415, has been confirmed by different health authorities [49]. Moreover, EFSA [33] has developed some guidelines for the safety assessment of *E. faecium* strains intended for feed supplementation. With the major focus thus far on the probiotic potential and safety of *E. faecalis* and *E. faecium* strain, strains of the species *E. durans*, *E. hirae* and *E. mundtii* have also been suggested for their beneficial functions and safety [50]. The presence of *Enterococcus* species (Table 1) different from those indicated on the product labels may be explained either by wrong classification or by contamination. The latter may be the case for those products where no enterococci were declared; particularly in these cases such contaminants are of special concern due to their potentially opportunistic nature.

*Staphylococcus epidermidis* belongs to the normal skin microbiota of the human host. This species was detected in one out of three batches of product H using 3M Staph Express Petrifilm™ with levels of approximately 10^3^ CFU/g. This may indicate poor quality control during production and possibly reflects negligence in the observation of Good Microbiological Practices during the manufacturing process. The detection of *Staphylococcus* species in probiotic products has been formerly reported [19,51]. Although *Staph. epidermidis* belongs to the normal skin and nasopharyngeal microbiota, it may be involved in opportunistic infections and may pose a risk for immuno-compromised patients [52,53]. The presence of any potentially opportunistic pathogen in a probiotic product constitutes a health hazard; this is of special concern for infants because of an elevated risk of colonization and persistence in the GIT [54,55]. Several examples of mislabeling and nomenclatural mistakes have been reported for commercial probiotics in the recent years [19,51,56,57].

### 3.3. Metagenomic Analyses of Probiotic Products by Next Generation Sequencing

Application of different approaches is important to provide substantial evidence of the exact microbiological quality of the evaluated probiotic products since each method has its own strengths and limitations. Modern biomolecular approaches are considered appropriate in the determination of the microbial diversity within the probiotic products and precision to estimate proportions and levels of bacterial counts. However, the cost of these analyses is still high and the procedures generally require expensive equipment and highly skilled laboratory personnel. Metagenomic evaluation has been increasingly used to study the complex microbial content of the GIT. It is proven as a useful tool since it can provide a bigger picture of not only the dominant bacteria but even the minority present in a sample, including uncultured bacterial species. Results of the metagenomic evaluation of products A and G are summarized in Table 2. Product A was of particular interest due to the high differences between detected culturable bacterial species and those claimed in the product specifications, indicating the presence of 12 different species (Table 1). Furthermore, this product had the largest difference between the claimed and observed total bacterial counts (Figure 1). Based on the performed metagenomic analysis, the microorganisms detected on the phylum level in product A belonged to the Proteobacteria, Firmicutes and Actinobacteria, while the presence of Firmicutes and Actinobacteria was confirmed for product G. Quantitative analysis of product A on the genus level showed *Bifidobacterium* to represent 28.2% of the population, 5.1% for *Bacillus*, 43.6% for *Enterococcus*, 7.7% for *Lactobacillus*, 5.1% for *Delftia* and 10.3% for an unknown genus of the *Enterobacteriaceae*. *Delftia* spp. and *Bacillus* spp. were not declared on the specification of product A. On the other hand, the metagenomic analysis showed *Bifidobacterium* to represent 69.6% of the population in product G and the remaining 30.4% to be *Enterococcus. Lactobacillus* spp. was not detected by metagenomic analysis; this may be due to the specificity of the primers used. These results demonstrate a huge discrepancy between the product declaration and the actual microbial population recovered from these products.

### 3.4. Safety Evaluation of the Isolates

The culture-independent and dependent analyses of the microbial consortia showed the presence of *Enterococcus* spp. in most of the 10 probiotic products without any indication on the label. As previously discussed, *Enterococcus* spp. can play a beneficial role in different fermented food products [58] while some strains are applied as probiotics [59]. Yet, several *Enterococcus* spp. are described as opportunistic pathogens [60]. In addition, in the genus *Streptococcus* only a few species are generally recognized as safe (GRAS) by FDA, comprising *Streptococcus thermophilus* [61], *Streptococcus gallolyticus* subsp. *macedonicus* and *Streptococcus salivarius* subsp. *salivarius* [62]. The safety of all other *Streptococcus* spp. requires careful assessment. Appropriate safety tests are therefore essential before suitability declaration of specific strains of *Enterococcus* spp. and *Streptococcus* spp. for the human and animal applications.

#### 3.4.1. Hemolysis Activity of the Isolates

Most of the microorganisms recovered from the ten commercial probiotic products exhibited no (gamma) hemolysis when streaked on blood agar plates. β-hemolysis was detected for only isolate, *Staph. epidermidis* HEM C56, from one batch of product H. Moreover, this isolate (*Staph. epidermidis*) cannot be considered as a typical component of probiotic preparations, and its presence most probably reflects contamination. Hemolytic activity is considered an important virulence factor in pathogenesis and facilitates acquisition of iron to the pathogen, thereby leading to conditions such as anemia or oedema in the host [63]. No β-hemolysis positive strains should be present in any probiotic formula or fermented food products. Being a health risk to adult consumers their presence may be more serious for infants and children with a lesser-developed immune system.

#### 3.4.2. Detection of Biogenic Amines Produced by the Isolates

Biogenic amines (BA) are bioactive compounds with low molecular weight and are products of decarboxylation of precursor amino acids. Most significant BAs are histamine, tyramine, putrescine and cadaverine which is derived from histidine, tyrosine, ornithine and lysine respectively. BAs are associated with food spoilage and fermentation process [64]. Hence, bacteria involved in fermentation such as lactic acid bacteria can produce BAs either as a response to sugar depletion and excessive acid levels [65]. Among the four BAs, histamine and tyramine are considered the highest health risk due to severity of symptoms such as “scromboid fish poisoning” and “cheese reaction” [66]. The first one is known to be associated with consumption of spoiled fish with high levels of histamine and may cause symptoms such as flushing of the face, abdominal pain, diarrhea, headache and palpitations [67]. The term cheese reaction is linked to consumption of cheese with high levels of tyramine. Symptoms may range from hypertension, migraine, headaches and possibly other neurological complications [68]. A study made by Linares et al. [69] revealed that tyramine causes necrosis in HT29 intestinal cells while histamine induces apoptosis. Results for the biogenic amine production test of obtained isolates were summarized in Table 3. *Bac. coagulans* HEM C18 from product A produced tyramine and histamine. Tyramine production were observed to be produced by some *Enterococcus* spp. both declared and undeclared in the product labels particularly *E. faecalis* HEM C48, undeclared on the label from Product E and *E. faecium* from Product G. Enterococci are known to commonly produce tyramine. *E. faecium* and *E. faecalis* have been observed to accumulate tyramine during their late exponential phase. This suggests that enterococci produce tyramine not as a response to nutrient depletion but as a natural phenomenon since tyrosine decarboxylase is secreted by enterococci outside of their cell environment [70]. The ability to produce tyramine is found to be species specific to *E. faecalis* but may also be common among strains of *E. faecium* and *E. durans* [71]. Isolated *Staph. epidermis* HEM C56 from Product H produced putrescine. Coton et al. [72] showed that some coagulase-negative staphylococci such as *Staph. epidermidis* may have a specific pathway for production of putrescine.

In this study, BA production was assessed qualitatively by observing color change of the decarboxylase media from yellow to purple indicating increase in pH. Other methods such as High Performance Liquid Chromatography (HPLC) that can quantify BA production may also be used to further assess the safety of microorganisms in probiotic products [73]. EFSA recommends 50 mg histamine for healthy individuals and 600 mg tyramine for healthy individuals except those hypertensive individuals taking monoamine oxide inhibitor (MOI) drugs [74]. No established levels of putrescine and cadaverine have been set because their exact adverse health effects are not yet known. However, these BAs have been shown to increase the harmful effects of histamine.

#### 3.4.3. Antibiotic Resistance of the Isolates

The MIC values of each isolate from commercial probiotic products were listed in Table 3. Among the three microorganisms from Product A, *Bac. coagulans* HEM C18 was resistant to vancomycin as per the EFSA recommended MIC values. *E. faecium* HEM C143 and *L. plantarum* HEM C163 from product A were both resistant to chloramphenicol, clindamycin, erythromycin, gentamicin and kanamycin. *L. plantarum* HEM C163 was also tetracycline resistant. From Product B, *Strep. thermophilus* HEM C52 and *L. gallinarum* were susceptible to all nine antimicrobials, while Product B *L. rhamnosus* HEM C14 was resistant to several antimicrobials including ampicillin, kanamycin and streptomycin. Other isolates from Product B, *L. paracasei* HEM C39 and *L. acidophilus* HEM C16 have higher MIC values compared to EFSA cut-off for kanamycin and chloramphenicol respectively. *L. reuteri* HEM C1 from Product C exhibited resistance to ampicillin and tetracycline. The *E. durans* contaminant recovered from one batch of Product C was resistant to chloramphenicol, clindamycin, erythromycin, kanamycin and gentamicin. Product D *L. rhamnosus* HEM C3 and *L. acidophilus* HEM C21 from Product E were susceptible to all nine antibiotics. *E. faecalis* HEM C48 though not listed on the label of Product E, was also susceptible to the tested antibiotics. *L. plantarum* from Product F was resistant to tetracycline. *L. plantarum* HEM C25, although undeclared on the label of Product G was susceptible to the nine antibiotics. However, *E. faecium* HEM C22 from Product G was resistant to erythromycin. *Bif. breve* HEM C28 from Product G was only susceptible to chloramphenicol and gentamicin and were resistant to the other antibiotics. Two isolates from Product H, *L. plantarum* HEM C33 and *Strep. thermophilus* HEM C31 were susceptible to the nine antibiotics. Isolated *Bif. breve* HEM C30 from Product H was resistant to erythromycin, gentamicin, kanamycin and vancomycin whereas *E. faecium* HEM C54 not listed as part of its formulation was resistant to erythromycin, kanamycin and streptomycin. Only *Bac. coagulans* HEM C136 and *E. faecium* HEM C128 from Product I showed resistance to most antibiotics tested except for tetracycline and vancomycin. *L. reuteri* HEM C79 from Product I was resistant to chloramphenicol. In contrast, *L. reuteri* HEM C148, the only microorganism recovered from Product J that matched its label was susceptible to all nine antibiotics. Conversely, isolates *L. sakei* HEM C81, *E. faecium* HEM C100 and *E. durans* HEM C64 were resistant to at least two antibiotics.

Most of *Lactobacillus* spp. and *Bifidobacterium* spp. being used as probiotics have a long history of safe use and thus acquired the GRAS status. However, since there is a rising concern of antibiotic resistance and probiotics have been used before to replenish the gut microbiota after a course of treatment with antibiotics, there is a vast source of antibiotic resistance genes present in the gut [75]. Antibiotic resistance in itself is not a virulence factor, but the risk of possible horizontal gene transfer of antibiotic resistance genes, specially that located on plasmids from probiotic microorganisms to pathogens could be a serious risk [76]. A study by Mater et al. [77] demonstrated that vancomycin resistance of two strains of vancomycin-resistant enterococci can be transferred to a commercial probiotic *L. acidophilus* strain both in vitro and in vivo during digestion in mice in high rates. Therefore, checking for the antibiotic resistance of commercial probiotic strains is important. Similar studies have checked commercial probiotic products for antibiotic resistance and observed that some strains used in the probiotic formulations have resistance to some antibiotics [75,78,79]. It is essential though to further check if the observed resistance is intrinsic (inherent) or extrinsic (acquired) since there will be higher risk of horizontal transfer from microorganisms with acquired antibiotic resistance. Transferability of antibiotic resistance and presence of other potential virulence factors such as lecithinase and gelatinase should also be checked by the manufacturer before marketing of a strain.

### 3.5. Simulated Stomach-Duodenum Passage

In order to contribute to the well-being of the host, a sufficient number of probiotics have to survive the conditions of the stomach and upper part of the GIT and reach the appropriate parts of the GIT. An in vitro model using MRS pH 2.5 with the addition of oxgall and synthetic duodenum juice was used in the study to assess if the probiotic isolates could possibly survive up to the duodenum [80].

In the study, the different LAB isolated from commercial probiotic products exhibited different levels of survival in the simulated passage through the stomach and duodenum. As shown in Table 4 among the *Enterococcus* species recovered and declared in the product labels, the *E. faecium* strain from product A had the highest survival (45.79%) after passage through the simulated duodenum conditions. However, *Enterococcus* spp. from other products (Products G and J) had lower survivability in the duodenum test. The difference in performance could be the origin of isolation of the *Enterococcus* species. *E. faecium* isolated from human gut was observed to survive low pH and bile salts [81]. *L. reuteri* from different products (Products C, I and J) performed differently in the SSDP test with 0.016%, 0.022% and 0.226% respectively. Same was observed in different *L. plantarum* strains from Product A having the highest observed survival rate of 67.97% in the SSDP test and *L. rhamnosus* strains. *L. acidophilus* from both products B and E had a high survival in acid after 1 h incubation with 100 and 131 percent survival respectively. *L. acidophilus* NCFM strain is a known probiotic found in various commercial probiotics for humans. Improvement of survival of *L. acidophilus* in low pH was demonstrated by addition of trehalose [82]. However, both strains of *L. acidophilus* from product B and E had less than one percent survival after the oxgall and duodenum stress. The *Bif. breve* strain from Product G had lower than detectable limits (1 × 10^5^ CFU/mL) after pH stress and had less than one percent survivability in oxgall and duodenum. Similar observations were seen from the *Bif. breve* isolated from Product H. Some mechanisms how the LAB may survive the upper GIT have been proposed. First, is the maintenance of the internal pH in fermentative bacteria like LAB. A possible pH stress mechanism is the presence of F_0_F_1_-ATPase proton pumps which actively pumps out H+ protons to keep the internal pH inside the bacterial cell [83]. Secondly, one of the biggest hurdles of the upper GIT is the presence of bile salts. One factor that affects their survival is the presence of bile salt dehydrolases which can disrupt the lytic effect of bile salts [84].

### 3.6. Antagonistic Activity of the Isolates Against Common GIT Pathogens

Probiotics are often used as “dietary supplements to improve gut health and prevent enteric infections”. To be able to confer benefits to the host, they must be able to maintain microbial balance in the human gut. They are often taken to control foodborne illness through different mechanisms such as competitive exclusion against GIT pathogens [85].

The obtained isolates were evaluated for potential antagonistic activity against some GIT-related pathogens such as *Bac. cereus* ATCC 11778, *Staph. aureus* subsp. *aureus* ATCC 6538, *Escherichia coli* ATCC 8739, *Listeria innocua* ATCC 33090 and *Salmonella enterica* subsp. *enterica* serovar Typhimurium ATCC 14028. Cell-free supernatants were initially used but did not exhibit any antagonistic activity against the pathogens. Eighteen-hour bacterial cultures were used instead.

As shown in Table 5, *Bac. cereus* ATCC 11778 was inhibited by only two strains (*L. rhamnosus* HEM C3 from product B *and L. sakei* HEM C61 from product I), and *Staph. aureus* ATCC 6538 by *L. plantarum* C25, *E. faecium* HEM C22 from product G and *L. sakei* HEM C61 from product I. This *L. sakei* strain (HEM C61) and *L. plantarum* HEM C25 from product G also inhibited the growth of *Sal. enterica* ATCC 14028 but not of *E. coli* ATCC 8739. Inhibition may be attributed mainly to acid production resulting in pH reduction, with overnight cultures reaching pH-values between 3.5 and 5.0. Mechanisms underlying the observed antagonistic activities have not been investigated further probably, and, in addition to acid formation, these may be linked to the production of different antimicrobial metabolites such as bacteriocins, diacetyl, carbon dioxide, hydrogen peroxide, low molecular antimicrobials, phenyl lactic and acetate [86]. Intrinsic can extrinsic factors may play a role in (e.g.,) bacteriocin production, and temperature-dependently some *Lactobacillus* spp. have been shown to produce higher levels of bacteriocin at 30 °C than at 37 °C [87,88].

Without international consensus on standard criteria for quality and safety evaluation of commercial probiotics, it is difficult to make a final assessment on the products investigated in this study. This investigation could be considered as a model study to obtain scientific information on probiotic products intended for infants and young children. For this population group, the potential therapeutic value of probiotics (also including pre- and synbiotics) for stabilizing the gut microbiota of infants and children is recognized [89]. Yet, information on optimal therapeutic applications probiotics, is still deficient [90]. In fact, clear guidelines on strain selection, mode and dose of application, and criteria for safety testing, amongst others, have not been established yet. In particular, well proven safety of a probiotics should be established before its administration during infancy [91].

Meanwhile, important steps towards “global harmonization” have been taken when the Codex Alimentarius Commission’s Committee on Nutrition and Foods for Special Dietary Uses, under the Joint FAO/WHO Food Standards Programme, have convened in Düsseldorf, Germany, from 24–29 November 2019 [92]. In its function under the World Trade Organization (WTO) the Codex Alimentarius Commission “develops and adopts food standards that serve as a reference for international food trade“. Contributions by governmental and non-governmental organizations (NGO’s) on pre-, pro- and synbiotics are likewise coordinated by the Codex Alimentarius Commission. Based on their Singapore meeting of June 2018, the International Scientific Association of Probiotics and Prebiotics (ISAPP) prepared a white paper suggesting “minimum criteria for harmonizing global regulatory approaches for probiotics in foods and supplements” (https://4cau4jsaler1zglkq3wnmje1-wpengine.netdna-ssl.com/wp-content/uploads/2018/10/ summary-document-probiotics-criteria-ISAPP.pdf). This paper was “intended to provide perspective to local regulators for discussions on this topic” at the subsequent meeting of the Codex Alimentarius Commission in 2019. A major objective was to obtain (international) agreement on minimum standards for pro- and prebiotics. These activities have been preceded by several measures taken by different countries towards defining criteria for probiotics. Examples are the regulatory categorization of probiotics [93] and the issuing of regulations governing label claims for food products, including probiotics [94] by the US Food and Drug Administration. Moreover, and as example, a Task Force of the Indian Council of Medical Research has proposed guidelines for the evaluation of probiotics in food in 2011 [95]. The importance of correct labelling has been exemplified by the outcome of this study. This includes discrepancies in the matching of detected microbial numbers organisms with those declared on labels of some products. An issue of concern is the contamination of 4 out of 10 products with *Enterococcus* strains (not declared on the labels), some of which exhibited resistance to clindamycin. Moreover, the detection of a ß-hemolytic *Staph. epidermidis* strain as presumed contaminant in one product may have constitute a health risk, in particular to infants.

## 4. Conclusions

According to the authors’ best knowledge this was the first study assessing the quality of some probiotic products intended for children and marketed in the Republic of the Philippines and the Republic of Korea. The procedures we used to evaluate these products included viable counts, molecular identification, safety, survival in simulated stomach-duodenum passage and antimicrobial properties.

With this study the authors obtained some insight into the quality of some commercial probiotics intended for children. While international harmonization on quality and safety probiotics has not been achieved yet, shortcomings of some of the investigated products are obvious and specifically include the presence of contaminating strains such as enterococci and a ß-hemolytic strain of *Staph. epidermidis*. There seems to be an urgent need for international consensus on guidelines both for industry and governments/regulating as a basis for regulating commercial probiotic products. This will contribute to increased confidence of consumers in the (claimed) benefits of these products. In addition, commercialization of new probiotics should be carefully regulated and in accordance with international standards.

## Figures and Tables

**Table 1:** Microorganisms listed on the label claims of probiotic products for children compared to those recovered and identified based on culture-dependent identification (16S rRNA sequencing).

Product	Label Claim	Species Identified Using 16S rRNA Sequencing		
	Bacterial Species Declared	Identified Microorganisms from Probiotic Products	Assigned Strain Number	Phylogenetic Tree Showing Relationship of Microorganisms Isolated from Each Probiotic Product
A	*Lactobacillus plantarum*	*Lactobacillus plantarum* ^#^	HEM C163	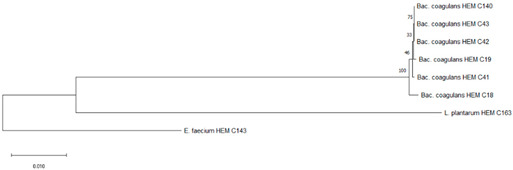 Phylogeny of isolated microorganisms from product A based on 1466 nucleotides of the partial 16S rRNA gene using the neighbor-joining method. The sum of branch length = 0.17651297 is shown. Scale bar represents one nucleotide substitution for every 100 nucleotides. †
	*Bifidobacterium breve* subsp. *breve*	*Bacillus coagulans*	HEM C18	
	*Bifidobacterium infantis* subsp. *infantis*		HEM C19	
	*Bifidobacterium longum*		HEM C41	
	*Enterococcus faecalis*		HEM C42	
	*Lactobacillus acidophilus*		HEM C43	
	*Lactobacillus brevis*		HEM C140	
	*Lactobacillus bulgaricus*	*Enterococcus faecium*	HEM C143	
	*Lactobacillus casei*			
	*Lactobacillus fermentum*			
	*Lactobacillus helveticus* subsp. *jugurti*			
	*Streptococcus thermophilus*			
B	*Streptococcus thermophilus*	*Streptococcus thermophilus* ^#^	HEM C52	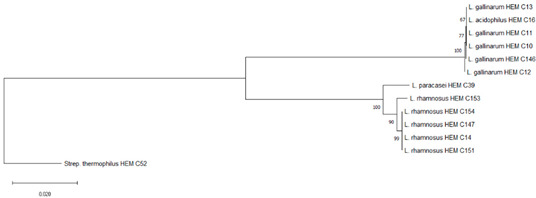 Phylogeny of isolated microorganisms from product B based on 1445 nucleotides of the partial 16S rRNA gene using the neighbor-joining method. The sum of branch length = 0.22020732 is shown. Scale bar represents two nucleotide substitutions for every 200 nucleotides. †
	*Lactobacillus acidophilus*	*Lactobacillus acidophilus* ^#^	HEM C16	
	*Lactobacillus rhamnosus*	*Lactobacillus rhamnosus* ^#^	HEM C14	
	*Lactobacillus casei*		HEM C147	
	*Lactobacillus bulgaricus*		HEM C151	
	*Bifidobacterium breve*		HEM C153	
	*Bifidobacterium infantis*			
		*Lactobacillus paracasei*	HEM C39	
		*Lactobacillus gallinarum*	HEM C10	
			HEM C11	
			HEM C12	
			HEM C13	
			HEM C146	
			HEM C52	
C	*Lactobacillus reuteri*	*Lactobacillus reuteri* ^#^	HEM C1	
		*Enterococcus durans*	HEM C121 *	
D	*Lactobacillus rhamnosus*	*Lactobacillus rhamnosus* ^#^	HEM C3	
E	*Lactobacillus acidophilus*	*Lactobacillus acidophilus* ^#^	HEM C21	
	*Bifidobacterium longum*	*Enterococcus faecalis*	HEM C48	
F	*Lactobacillus plantarum*	*Lactobacillus plantarum* ^#^	HEM C5	
G	*Enterococcus faecium*	*Enterococcus faecium* ^#^	HEM C22	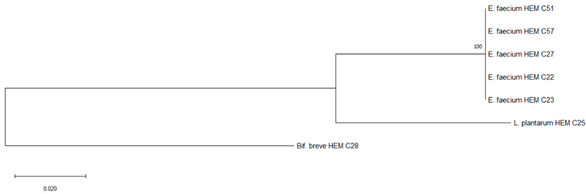 Phylogeny of isolated microorganisms from product G based on 1390 nucleotides of the partial 16S rRNA gene using the neighbor-joining method. The sum of branch length = 0.26710396 is shown. Scale bar represents two nucleotide substitutions for every 200 nucleotides. †
	*Bifidobacterium longum*		HEM C23	
	*Bifidobacterium bifidum*		HEM C27	
	*Lactobacillus acidophilus*		HEM C51	
			HEM C57	
		*Bifidobacterium breve*	HEM C28	
		*Lactobacillus plantarum*	HEM C25	
H	*Streptococcus thermophilus*	*Streptococcus thermophilus* ^#^	HEM C31	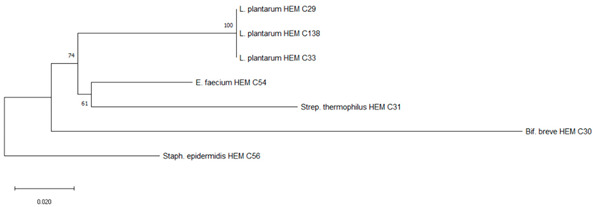 Phylogeny of isolated microorganisms from product H based on 1376 nucleotides of the partial 16S rRNA gene using the neighbor-joining method. The sum of branch length = 0.39666877 is shown. Scale bar represents two nucleotide substitutions for every 200 nucleotides. †
	*Bifidobacterium breve*	*Bifidobacterium breve* ^#^	HEM C30	
	*Lactobacillus plantarum*	*Lactobacillus plantarum* ^#^	HEM C29	
	*Bifidobacterium longum*		HEM C33	
			HEM C138	
		*Enterococcus faecium*	HEM C54	
		Staphylococcus epidermidis *	HEM C56	
I	*Lactobacillus sporogenes*	*Bacillus coagulans* ^#^	HEM C136	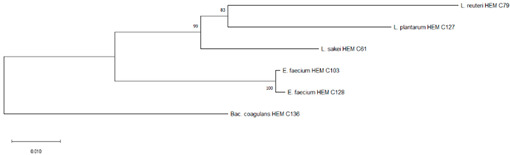 Phylogeny of isolated microorganisms from Product I based on 1371 nucleotides of the partial 16S rRNA gene using the neighbor-joining method. The sum of branch length = 0.23418728 is shown. Scale bar represents one nucleotide substitutions for every 100 nucleotides. †
	*Lactobacillus johnsonii*	*Lactobacillus sakei*	HEM C61	
	*Lactobacillus rhamnosus*	*Lactobacillus reuteri*	HEM C79	
	*Bifidobacterium lactis*	*Enterococcus faecium*	HEM C103	
			HEM C128	
		*Lactobacillus plantarum*	HEM C127	
J	*Lactobacillus reuteri*	*Lactobacillus reuteri* ^#^	HEM C65	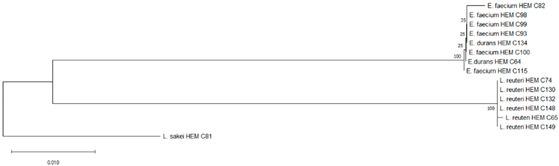 Phylogeny of isolated microorganisms from product I based on 1385 nucleotides of the partial 16S rRNA gene using the neighbor-joining method. The sum of branch length = 0.13083425 is shown. Scale bar represents one nucleotide substitutions for every 100 nucleotides. †
	*Lactobacillus acidophilus*		HEM C74	
	*Bifidobacterium bifidum*		HEM C130	
	*Bifidobacterium animalis* subsp. *lactis*		HEM C132	
	*Streptococcus thermophilus*		HEM C148	
	*Enterococcus faecium*		HEM C149	
		*Enterococcus faecium* ^#^	HEM C82	
			HEM C93	
			HEM C98	
			HEM C99	
			HEM C100	
			HEM C115	
		*Enterococcus durans*	HEM C64	
			HEM C134	
		*Lactobacillus sakei*	HEM C81	

Species identified by culture-dependent methods but not included on the label were given strain numbers as deposited in the HEM culture collection. n.d.— not declared; # presumed to be the strain indicated on the label; * detected in one batch of the product. † The “evolutionary” history was inferred using the Neighbor-Joining method [42]. The optimal tree with the sum of branch lengths is shown for each product. The percentage of replicate trees in which the associated taxa clustered together in the bootstrap test (10,000 replicates) is shown next to the branches [25]. The tree is drawn to scale, with branch lengths in the same units as those of the evolutionary distances used to infer the phylogenetic tree. The evolutionary distances were computed using the p-distance method [43] and are in the units of the number of base differences per site. All ambiguous positions were removed for each sequence pair (pairwise deletion option). Evolutionary analyses were conducted in MEGA X [24].

**Table 2:** Bacterial composition of products A and G based on their label claims, determined by culture-dependent 16S rRNA sequencing and metagenomic analyses of the 16S rRNA gene.

	Culture-Independent (Metagenomic Analysis)			
Product	Phylum	Relative Abundance	Genera	Relative Abundance
A	Proteobacteria	15.4%	Unidentified genus	10.3%
			*Delftia*	5.1%
	Firmicutes	56.4%	*Lactobacillus*	7.7%
			*Enterococcus*	43.6%
			*Bacillus*	5.1%
	Actinobacteria	28.2%	*Bifidobacterium*	28.2%
G	Firmicutes	30.4%	*Enterococcus*	30.4%
	Actinobacteria	69.6%	*Bifidobacterium*	69.6%

**Table 3:** Hemolysis, biogenic amine production, antibiotic resistance and minimum inhibitory concentrations of the isolated bacterial species in commercial probiotic products for children. Strains have been deposited in the internal collection under the numbers shown in the table.

					Minimum Inhibitory Concentration (mg/L)									
Product	Isolate	Strain	Hemo-lysis	BA Produced	AMP	CHL	CLI	ERY	GEN	KAN	STR	TET	VAN	
A	*Bac. coagulans*	HEM C18	γ	Tyr, His	n.r.	1	≤0.25	≤0.25	1	8	4	≤1	16 *	
	*E. faecium*	HEM C143	γ	None	2	32 *	>16 *	16 *	64 *	>1024 *	128	≤1	2	
	*L. plantarum* ^#^	HEM C163	γ	None	2	16 *	8 *	2*	32 *	>128 *	n.r.	>128 *	n.r.	
B	*L. rhamnosus* ^#^	HEM C14	γ	None	16 *	1	≤0.50	0.5	16	>128 *	>128 *	2	n.r.	
	*L. acidophilus* ^#^	HEM C16	γ	None	≤0.25	8*	0.5	≤0.25	≤8	32	≤8	4	1	
	*L. paracasei*	HEM C39	γ	None	1	2	≤0.5	≤0.25	32	>128 *	64	4	n.r	
	*L. gallinarum*	HEM C9	γ	None	≤0.25	1	≤0.25	≤0.25	1	32	≤2	≤1	1	
	*Strep. thermophilus* ^#^	HEM C52	γ	None	≤0.50	4	≤0.50	≤0.50	2	32	≤2	≤1	≤1	
C	*L. reuteri* ^#^	HEM C1	γ	None	>8 *	2	≤0.25	≤0.25	1	32	8	>64 *	n.r.	
	*E. durans*	HEM C121	γ	None	1	64 *	>16 *	8 *	64 *	>1024 *	128	≤1	2	
D	*L. rhamnosus* ^#^	HEM C3	γ	None	1	1	≤0.50	≤0.25	8	64	≤32	2	n.r	
E	*L. acidophilus* ^#^	HEM C21	γ	None	0.5	1	≤0.25	≤0.25	4	64	4	4	≤0.50	
	*E. faecalis*	HEM C48	γ	Tyr	≤0.25	1	≤0.25	≤0.25	4	64	4	2	2	
F	*L. plantarum* ^#^	HEM C5	γ	None	1	1	≤0.50	≤0.25	8	128 *	n.r.	64 *	n.r	
G	*L. plantarum*	HEM C25	γ	None	1	8	0.5	≤0.25	≤2	≤16	n.r.	32	n.r	
	*E. faecium* ^#^	HEM C22	γ	None	1	≤4	≤1	16 *	32	512	128	≤1	≤1	
	*Bif. breve*	HEM C28	γ	None	16 *	8	>8 *	>8 *	64	n.r.	64	1	>16 *	
H	*Strep. thermophilus* ^#^	HEM C31	γ	None	≤0.50	2	≤0.50	≤0.50	≤1	8	≤2	≤1	≤1	
	*L. plantarum*	HEM C33	γ	None	1	8	0.5	≤0.25	≤2	≤16	n.r.	32	n.r	
	*E. faecium*	HEM C54	γ	None	1	≤4	≤1	8*	16	>1024 *	>128 *	≤1	≤1	
	*Bif. breve* ^#^	HEM C30	γ	None	2	8	>8 *	8	128*	n.r.	>128 *	2	>16 *	
	*Staph. epidermidis*	HEM C56	β	Put	n.d.	n.d.	n.d.	n.d.	n.d.	n.d.	n.d.	n.d.	n.d.	
I	*Bac. coagulans* ^#^	HEM C136	γ	None	n.r.	4	4	≤0.50	2	8	32 *	>32 *	≤1	
	*L. sakei*	HEM C61	γ	None	1	16 *	≤0.5	≤0.25	4	16	32	4	n.r.	
	*L. reuteri*	HEM C79	γ	None	1	16*	≤0.5	≤0.25	≤2	64	32	8	n.r.	
	*E. faecium*	HEM C128	γ	None	4 *	32 *	>16 *	16 *	64 *	>1024 *	128	≤1	4	
	*L. plantarum*	HEM C127	γ	None	1	8	≤0.25	≤0.25	≤2	64	n.r.	16	n.r.	
J	*L. reuteri* ^#^	HEM C148	γ	None	1	4	≤0.25	≤0.25	≤2	16	≤8	≤2	n.r.	
	*L. sakei*	HEM C81	γ	None	≤1	8 *	≤0.25	≤0.25	8	64	32	>32 *	n.r.	
	*E. faecium* ^#^	HEM C100	γ	None	1	8	>16 *	8 *	64 *	1024	128	≤1	2	
	*E. durans*	HEM C64	γ	None	1	8	>16 *	≤1	64 *	>1024 *	128	≤1	>16 *	
Controls	*Bacillus cereus*	ATCC 27348	β	n.d.	n.d.	n.d.	n.d.	n.d.	n.d.	n.d.	n.d.	n.d.	n.d.	
	*Escherichia coli*	ATCC 25922	α	His, Put, Tyr, Cad	n.d.	n.d.	n.d.	n.d.	n.d.	n.d.	n.d.	n.d.	n.d.	
	*Lactobacillus plantarum*	299V	γ	None	n.d.	n.d.	n.d.	n.d.	n.d.	n.d.	n.d.	n.d.	n.d.	
EFSA Cut-off Values	*Bacillus* spp.				n.r	8	4	4	4	8	8	8	4	
	*Strep. thermophilus*				2	4	2	2	32	64	64	4	4	
	*Bifidobacterium* spp.				2	4	1	1	64	n.r	128	8	2	
	*E. faecium*				2	16	4	4	32	1024	128	4	4	
	*L. plantarum*				2	8	2	1	16	64	n.r	32	n.r	
	*L. acidophilus/L. gallinarum*				1	4	1	1	16	64	16	4	2	
	*L. reuteri*				2	4	1	1	8	64	64	16	n.r	
	*L. rhamnosus*				4	4	1	1	16	64	32	8	n.r	
	*L. sakei*				4	4	1	1	16	64	64	8	n.r	

His: histamine; Put: putrescine; Tyr: tyramine; Cad: cadaverine. AMP: ampicillin; CHL: chloramphenicol; CLI: clindamycin; ERY: erythromycin; GEN: gentamicin; KAN: kanamycin; STR: streptomycin; TET: tetracycline; VAN: vancomycin; n.r.: not required. # presumed to be the strain indicated on the label; n.d.: not determined; * Strains with antibiotic resistance; cut-off values were established by the European Committee on Antimicrobial Susceptibility Testing (EUCAST, http://www.eucast.org/), EFSA.

**Table 4:** Survival of different microorganisms isolated from commercially available probiotic products in simulated stomach-duodenum passage test. For strain numbers refer to Table 3.

Product	Strains Isolated from Probiotic Products	Initial Counts	After 1 h (Stomach)		After 3h (Duodenum)	
		(Log CFU/mL)	(Log CFU/mL)	Survival %	(Log CFU/mL)	Survival %
A	*E. faecium* HEM C143 *	8.77 ± 0.04	8.62 ± 0.08	72.22	7.43 ± 0.04	45.79
	*L. plantarum* HEM C163	9.41 ± 0.07	9.33 ± 0.04	84.48	8.77 ± 0.07	67.97
B	*L. rhamnosus* HEM C14	8.67 ± 0.01	8.02 ± 0.03	22.60	3.15 ± 0.25	0.002
	*L. acidophilus* HEM C16	9.29 ± 0.05	9.29 ± 0.12	100.05	3.88 ± 0.62	0.00077
	*L. paracasei* HEM C39 *	9.42 ± 0.07	9.16 ± 0.27	59.43	3.09 ± 0.30	0.000056
	*Strep. thermophilus* HEM C52	9.98 ± 0.03	9.69 ± 0.04	51.72	8.31 ± 0.01	2.17
C	*L. reuteri* HEM C1	9.34 ± 0.20	9.34 ± 0.15	101.53	5.53 ± 0.21	0.016
D	*L. rhamnosus* HEM C3	9.20 ± 0.18	8.70 ± 0.08	35.08	<3.49	<0.001
E	*L. acidophilus* HEM C21	8.74 ± 0.06	8.83 ± 0.18	131.67	5.21 ± 0.17	0.0031
F	*L. plantarum* HEM C5	9.53 ± 0.07	9.49 ± 0.08	91.61	8.31 ± 0.02	25.81
G	*E. faecium* HEM C22	8.83 ± 0.17	8.58 ± 0.15	64.98	5.88 ± 0.06	0.011
	*L. plantarum* HEM C25	9.04 ± 0.37	8.58 ± 0.15	10.58	7.34 ± 0.22	1.011
	*Bif. breve* HEM C28 *	9.15 ± 0.00	<5.00	<0.10	4.21 ± 0.17	0.000122
H	*L. plantarum* HEM C33	9.36 ± 0.00	9.16± 0.11	77.81	7.49 ± 0.21	1.96
	*Bif. breve* HEM C30	8.81 ± 0.05	6.59 ± 0.16	0.61	5.60 ± 0.05	0.063
	*Strep. thermophilus* HEM C31	9.78 ± 0.04	9.17 ± 0.00	26.01	7.47 ± 0.08	0.0495
I	*L. reuteri* HEM C79 *	9.30 ± 0.08	8.30 ± 0.09	77.81	5.73 ± 0.05	0.022
	*L. plantarum* HEM C127 *	9.48 ± 0.03	9.35 ± 0.17	78.29	6.37 ± 0.10	0.080
J	*E. faecium* HEM C99	9.01 ± 0.11	8.65 ± 0.11	44.98	6.71 ± 0.04	0.513
	*L. reuteri* HEM C148	9.38 ± 0.08	9.30 ± 0.09	87.50	6.73 ± 0.05	0.226

* not declared on the label.

**Table 5:** Zones of inhibition against five different pathogens exhibited by isolates from commercial products. For strain numbers refer to Table 3.

		Test Pathogen Strains				
Product	Isolates from Probiotic Products	*Staphylococcus aureus* subsp. *aureus* ATCC 6538	*Bacillus cereus* ATCC 11778	*Escherichia coli* ATCC 8739	*Listeria innocua* ATCC 33090	*Salmonella enterica* subsp. *enterica* var. TyphimuriumATCC 14028
A	*E. faecium* HEM C143 *	-	-	-	-	-
	*L. plantarum* HEM C163	-	-	-	-	-
B	*L. rhamnosus* HEM C14	-	-	-	-	-
	*L. acidophilus* HEM C16	+	-	-	-	+
	*L. paracasei* HEM C39 *	-	-	-	-	-
	*Strep. thermophilus* HEM C52	-	-	-	-	-
C	*L. reuteri* HEM C1	-	-	-	-	-
D	*L. rhamnosus* HEM C3	-	+	-	-	+
E	*L. acidophilus* HEM C21	-	-	-	-	-
F	*L. rhamnosus* HEM C5	-	-	-	-	-
G	*E. faecium* HEM C22	+++	-	-	+++	-
	*L. plantarum* HEM C25 *	+++	-	-	+++	++
	*Bif. breve* HEM C30 *	-	-	-	-	-
H	*L. plantarum* HEM C33	-	-	-	-	-
	*Bif. breve* HEM C30	-	-	-	-	-
	*Strep. thermophilus* HEM C31	-	-	-	-	-
I	*L. sakei* HEM C61 *	+++	++	-	-	++
	*L. reuteri* HEM C79 *	-	-	-	-	-
J	*E. faecium* HEM C99	-	-	-	-	-
	*L. reuteri* HEM C148	-	-	-	-	-

+ 1–3 mm zone of inhibition; ++ 4–6 mm zone of inhibition; +++ >6 mm zone of inhibition. * not declared in the label.
